# Lower-Limb Strength Symmetry, Postural Control, and Clinical Characteristics in Individuals with ACL Injury and Chronic Ankle Instability

**DOI:** 10.3390/jcm15207979

**Published:** 2026-10-15

**Authors:** Begüm Kara Kaya, Hikmet Ucgun, Ahmet Veliyev, Ramil Feyzulov, Necdet Demir

**Affiliations:** 1 Department of Physiotherapy and Rehabilitation (English), Faculty of Health Sciences, Biruni University, Istanbul 34015, Türkiye; bkara@biruni.edu.tr; 2 Department of Physiotherapy and Rehabilitation, Faculty of Health Sciences, Istanbul Atlas University, Istanbul 34408, Türkiye; 3 Department of Physiotherapy and Rehabilitation, Postgraduate Education Institute, Istanbul Atlas University, Istanbul 34408, Türkiye; 4 Milli İdman Tibb və Reabilitasiya İnstitutu, Baku AZ1110, Azerbaijan; 5 Department of Orthopedics and Traumatology, Biruni University Hospital, Istanbul 34295, Türkiye

**Keywords:** muscle strength, postural balance, ankle injuries, anterior cruciate ligament injuries, patient-reported outcomes

## Abstract

**Objective:** To investigate the associations between lower-limb muscle strength symmetry and postural control, determine whether lower-limb symmetry explains additional variance in dynamic postural control after adjustment for demographic characteristics and diagnostic group, and compare these outcomes among individuals with anterior cruciate ligament (ACL) injury, chronic ankle instability (CAI), and healthy controls. **Methods:** This cross-sectional study included 72 participants (mean age, 29.6 ± 5.9 years) equally distributed among the ACL, CAI, and healthy control groups. Isokinetic strength was assessed at 60°/s and higher joint-specific velocities (240°/s for the knee and 180°/s for the ankle), with LSI calculated from peak torque at 60°/s. Static and dynamic postural control were evaluated using the single-leg stance test and a computerized stabilometric platform. Pain intensity (VAS), kinesiophobia (Tampa Scale), physical activity (International Physical Activity Questionnaire–Short Form (IPAQ-SF)), and health-related quality of life (Short Form-36) were also assessed. Between-group comparisons, Spearman correlation, and hierarchical multiple regression analyses were performed. **Results:** Significant between-group differences were observed in all postural control outcomes, most lower-limb symmetry indices, pain intensity, kinesiophobia, physical activity, and patient-reported outcomes (all *p* < 0.05). Pairwise comparisons showed better eyes-open static balance in healthy controls than in both clinical groups, poorer eyes-closed static balance in ACL than in the other groups, and better dynamic postural control in controls than in both clinical groups and in CAI than in ACL. Lower-limb symmetry differences involved different pairwise contrasts among controls, CAI, and ACL depending on muscle group and testing velocity. Two nominal associations between lower-limb symmetry and balance did not remain significant after FDR correction. Adding diagnostic group significantly increased the explained variance in dynamic postural control (ΔR^2^ = 0.581, *p* < 0.001), whereas adding lower-limb symmetry indices did not (ΔR^2^ = 0.015, *p* = 0.583). The final model explained 68.4% of the variance (adjusted R^2^ = 0.638). **Conclusions:** Individuals with ACL injury and CAI demonstrated poorer postural control than healthy controls, alongside between-group differences in several lower-limb muscle strength symmetry measures. Associations between lower-limb muscle strength symmetry and postural control were not significant after FDR correction, and symmetry indices did not explain additional variance in dynamic postural control beyond demographic characteristics and diagnostic group.

## 1. Introduction

Balance is defined as the ability to maintain the body’s center of mass within the base of support and is a fundamental component of postural control [1]. It is regulated through the integration of multiple systems, including neuromuscular control, proprioception, visual input, and vestibular function [1,2]. Muscle strength represents one potential contributor to postural control; however, postural control emerges from the integration of sensory, neuromuscular, biomechanical, and cognitive processes and is influenced by individual, environmental, and task-related demands [1,3].

Lower-extremity muscle strength contributes to dynamic stability and functional performance, and its impairment may negatively affect postural control and increase injury risk [4,5]. In clinical and research settings, inter-limb strength symmetry is commonly quantified using the limb symmetry index (LSI); however, LSI reflects relative rather than absolute muscle capacity. An LSI close to 100% does not necessarily indicate normal strength, as bilateral weakness or impairment of the reference limb may mask absolute strength deficits [6,7]. Lower-extremity injuries are among the most common musculoskeletal conditions and are associated with substantial functional limitations, reduced physical performance, and long-term disability [8]. Anterior cruciate ligament (ACL) injury and chronic ankle instability (CAI) are two common conditions involving different joints of the lower extremity [9,10]. Although they differ in injury mechanism and anatomical involvement, both are associated with neuromuscular and postural-control impairments and reduced functional performance [11,12].

ACL injury and CAI share several functional consequences despite affecting different joints of the lower extremity, with both conditions being associated with altered proprioceptive function and impaired postural control [13,14]. However, potential mechanisms contributing to these impairments may differ between conditions. In ACL injury, altered knee joint stability, proprioceptive input, and arthrogenic quadriceps inhibition have been proposed as possible contributors to postural-control deficits [14]. In CAI, recurrent ankle sprains, delayed peroneal muscle activation [15], and altered distal sensorimotor control have similarly been proposed as potential contributors to postural-control deficits [13]. Furthermore, neuromuscular control training has been investigated as a strategy for preventing ankle sprains in athletes, highlighting the clinical relevance of neuromuscular function in ankle injury prevention [16]. In addition to involving different joints and injury mechanisms, ACL injury and CAI differ in their patterns of sensorimotor impairment and condition-specific rehabilitation approaches [17,18]. These similarities and differences provide a rationale for examining whether associations between lower-limb muscle strength symmetry and postural control vary by diagnostic group.

Previous studies have demonstrated that individuals with ACL injury and CAI exhibit impaired static and dynamic postural control compared with healthy controls [19,20]. Nevertheless, the factors associated with these impairments, including the potential contribution of lower-limb muscle strength symmetry, remain insufficiently understood. In particular, whether lower-limb muscle strength symmetry explains additional variance in postural control beyond demographic characteristics and diagnostic group remains unclear.

Objective assessments of postural control and lower-limb muscle strength symmetry provide complementary information on different aspects of lower-extremity function [2,20]. Patient-related factors, including pain, kinesiophobia, physical activity level, and health-related quality of life, may also provide clinically relevant information regarding functional status [21,22,23,24]. However, the relationships between these patient-related factors and postural control across different lower-extremity injury populations have not been well established.

Clarifying these relationships may improve the understanding of factors associated with postural control in individuals with lower-extremity injuries. Therefore, the primary aim of this study was to investigate the associations among lower-limb muscle strength symmetry, postural control, and clinical characteristics, and to determine whether lower-limb muscle strength symmetry explains additional variance in dynamic postural control after adjustment for demographic characteristics and diagnostic group. The secondary aim was to compare lower-limb muscle strength symmetry, postural control, and clinical outcomes among individuals with ACL injury, CAI, and healthy controls. We hypothesized that lower-limb muscle strength symmetry would be associated with postural control and would explain additional variance in dynamic postural control beyond demographic characteristics and diagnostic group.

## 2. Methods

### 2.1. Study Design

This cross-sectional observational study was conducted between August 2025 and March 2026 in accordance with the Declaration of Helsinki and approved by the Atlas University Non-Interventional Scientific Research Ethics Committee (approval no: 07/48, date: 28 July 2025). All participants provided written informed consent prior to participation. The study was registered at ClinicalTrials.gov (NCT07419750, 19 December 2025). The reporting of this study followed the STROBE statement for observational studies.

### 2.2. Sample Size Calculation

The sample size was determined with reference to effect sizes reported in a recent study with similar characteristics, in which large effect sizes (Cohen’s d = 1.47–1.91) were observed for differences in lower-extremity muscle strength and postural control between groups [25].

The a priori sample-size calculation was intended to support the primary between-group comparison across the three study groups. Given the large effects reported in the previous study, a conservative large-effect assumption (f = 0.40) was selected rather than directly converting the reported Cohen’s d values. The sample size was calculated using G*Power software (version 3.1), assuming a significance level of 0.05 and statistical power of 80%. Based on a one-way analysis of variance (ANOVA) design with three groups, the minimum required sample size was determined to be 66 participants, corresponding to at least 22 individuals per group.

### 2.3. Participants

Participants were recruited from individuals who applied to the Department of Orthopaedics and Traumatology at Biruni University Hospital in Istanbul, as well as from healthy volunteers residing in Istanbul who responded to study announcements shared via social media. Eligible participants were between 18 and 40 years of age. A total of 72 participants were included in the study, with 24 participants in each of the ACL injury, CAI, and healthy control groups.

Individuals in the ACL group were required to have a unilateral ACL injury diagnosed 6 to 18 months prior to participation, with confirmation by magnetic resonance imaging. Clinical examination also included the Lachman, anterior drawer, and pivot-shift tests, with findings graded on a 0–3 scale according to the degree of knee laxity/instability. In reconstructed cases, a hamstring tendon autograft was used. Participants in the CAI group were required to have a diagnosis of chronic ankle instability established 6 to 18 months prior, a history of at least two ankle sprains affecting the involved ankle, and ongoing symptoms of functional instability, such as giving way or perceived ankle instability. In addition, self-reported ankle instability was confirmed using the Cumberland Ankle Instability Tool (CAIT), with a score of <24 required for inclusion, in accordance with the International Ankle Consortium recommendations [26]. Healthy control participants had no history of lower extremity injury or orthopedic conditions.

All participants were required to be able to understand and communicate in Turkish and to provide written informed consent prior to participation. Participants with ACL injury or CAI had received conventional rehabilitation before study enrollment, as confirmed by self-report. Rehabilitation had been completed before enrollment, and no participant was receiving active treatment at the time of assessment. Detailed information regarding its duration, frequency, and content was not available. Diagnosis of ACL injury and chronic ankle instability was established by an orthopedic specialist who was part of the research team. Participants were excluded if they had any neurological, vestibular, or visual disorders that could affect balance performance, a history of other orthopedic conditions or trauma, musculoskeletal disorders or cardiovascular comorbidities, or any physiotherapy, rehabilitation, or surgical intervention within the previous six months.

### 2.4. Outcome Measures

Outcome measures included primary and secondary measures of balance performance, neuromuscular function, and patient-related parameters. Data were recorded using a standardized evaluation form developed by the research team. Assessments were performed by the same examiner under standardized conditions, with participants informed and familiarized with the procedures beforehand. Given the number of performance-based tests, the assessment battery was distributed across two visits, with adequate rest to minimize fatigue. The visits involved different components of the battery rather than repeated measurements of the same outcomes.

#### 2.4.1. Primary Outcome: Balance Assessments

Balance performance was evaluated under static and dynamic conditions. Static balance was assessed using a single-leg stance test under eyes-open and eyes-closed conditions. Participants stood barefoot on one leg with hands on the hips, and the duration of maintaining balance was recorded in seconds. Each trial was limited to a maximum of 150 s; no participant reached this ceiling. The test was terminated in case of loss of balance, including foot displacement, contact of the non-supporting limb with the ground, or use of arms for support. Three trials were performed for each limb, with a 2-min rest period between trials, and the best performance was used for analysis [27]. The uninvolved limb was tested first in the clinical groups, followed by the involved limb, whereas the dominant limb was tested first in healthy controls, followed by the non-dominant limb. Eyes-open testing was performed first, followed by eyes-closed testing. For between-group analyses, the involved limb was used in the ACL and CAI groups, whereas the dominant limb was used in healthy controls. A 10-min rest period separated the static and dynamic balance assessments to minimize potential fatigue.

Dynamic postural control was evaluated using a computerized stabilometric platform (ALFA, AC International East Sp. z o.o., Knurów, Poland; sampling frequency: 62 Hz), which analyzes center-of-pressure (COP) displacements based on ground reaction forces [28,29]. Assessments were performed using the platform’s integrated software and the Stability Limits Test protocol, with calibration conducted according to the manufacturer’s recommendations before testing. The platform was configured to allow movement in three planes with a total of eight degrees of freedom, and each assessment lasted 20 s. Participants stood barefoot on the platform and, without lifting their feet, controlled a cursor representing the COP by shifting their body weight toward 10 visually displayed target areas. These areas extended from the central Area 1 to progressively more peripheral areas up to Area 10, and the software recorded the proportion of the assessment spent within each area (%).

A weighted dynamic postural-control score was calculated as follows: Score = [(Area 1 × 10) + (Area 2 × 9) + (Area 3 × 8) + (Area 4 × 7) + (Area 5 × 6) + (Area 6 × 5) + (Area 7 × 4) + (Area 8 × 3) + (Area 9 × 2) + (Area 10 × 1)]/10. Thus, greater weighting was assigned to time spent in the more central target areas, and higher scores indicated better dynamic postural-control performance.

#### 2.4.2. Secondary Outcomes

##### Isokinetic Muscle Strength and Limb Symmetry Index

Isokinetic muscle strength was assessed using the Biodex System Pro 4 isokinetic dynamometer (Biodex Medical Systems Inc., Shirley, NY, USA), which has demonstrated generally good-to-excellent test–retest reliability for lower-limb measurements [30]. The quadriceps and hamstrings were assessed at the knee, and the dorsiflexors and plantarflexors at the ankle.

Following a standardized warm-up and familiarization procedure, knee testing was performed at 60°/s (4 repetitions) and 240°/s (20 repetitions), and ankle testing at 60°/s (10 repetitions) and 180°/s (15 repetitions). Participants were stabilized using the dynamometer straps to minimize compensatory movements, with standardized joint-specific positioning and range-of-motion settings. Gravity correction was performed according to the standardized Biodex procedure. Limb dominance in healthy controls was defined as the preferred leg for kicking a ball. The non-injured limb was tested first in the clinical groups, followed by the injured limb, whereas the dominant limb was tested first in healthy controls, followed by the non-dominant limb. The testing order was fixed rather than randomized, with a 3-min rest interval between tests to minimize potential fatigue.

The highest peak torque recorded across repetitions was used and normalized to body weight, expressed as Peak Torque/Body Weight (%). Inter-limb symmetry was quantified using the limb symmetry index (LSI) based on peak torque at 60°/s. LSI was calculated as (involved limb/uninvolved limb) × 100 for the ACL and CAI groups and (dominant limb/non-dominant limb) × 100 for healthy controls; thus, the reference limbs differed between the clinical and control groups.

The 60°/s condition was selected for the regression analysis because it provided a common low-velocity assessment across both knee and ankle muscle groups, whereas the higher-velocity protocols were joint-specific (240°/s for the knee and 180°/s for the ankle) and involved different repetition schemes. Using one LSI measure per muscle group at 60°/s also provided a consistent and parsimonious set of strength-symmetry variables, avoiding multiple velocity-specific measures of the same construct.

##### Pain Assessment

Pain intensity during activity was assessed using the Visual Analog Scale (VAS), a widely used measure of pain intensity with established validity and reliability [31]. Activity-related pain referred to pain experienced during usual daily functional activities, such as walking and stair negotiation, rather than during a standardized provocative task. Participants rated their pain on a 10-cm horizontal line ranging from “no pain” to “worst imaginable pain,” and scores were recorded in centimeters, with higher scores indicating greater pain intensity.

##### Kinesiophobia

Kinesiophobia was evaluated using the Tampa Scale of Kinesiophobia (TSK), a 17-item Likert-type questionnaire assessing fear of movement and reinjury. The Turkish version has demonstrated acceptable reliability (Cronbach’s alpha = 0.78) [32]. Total scores range from 17 to 68, with higher scores indicating greater levels of kinesiophobia.

##### Physical Activity Level

Physical activity level was assessed using the International Physical Activity Questionnaire-Short Form (IPAQ-SF), which evaluates the frequency and duration of physical activity performed over the previous seven days. The Turkish version of the IPAQ has demonstrated acceptable validity and reliability [33]. Total physical activity was expressed as metabolic equivalent (MET)-minutes per week, with higher scores indicating higher levels of physical activity.

##### Quality of Life

Quality of life was evaluated using the Short Form-36 (SF-36). The Turkish version of the SF-36 has been shown to be valid and reliable [34]. For the present study, the SF-36 Physical Health Summary and Mental Health Summary scores were calculated as the arithmetic mean of the corresponding subscale scores, each expressed on a 0–100 scale, with higher scores indicating better perceived health status.

### 2.5. Statistical Analysis

All statistical analyses were performed using IBM SPSS Statistics for Windows, version 24.0 (IBM Corp., Armonk, NY, USA). Continuous variables are presented as median [interquartile range (IQR)], whereas categorical variables are presented as frequency (n) and percentage (%). Normality of continuous variables was assessed using the Shapiro–Wilk test, supplemented by visual inspection of histograms and Q–Q plots. Shapiro–Wilk tests indicated normal distributions across all groups for dynamic postural control, quadriceps LSI at 60°/s and 240°/s, hamstring LSI at 240°/s, dorsiflexor LSI at 60°/s, and SF-36 Physical Health Summary; the remaining major outcomes showed departures from normality in at least one group.

Differences among the three groups were evaluated using the Kruskal–Wallis H test. When a significant overall group effect was identified, Mann–Whitney U tests were performed for post hoc pairwise comparisons, and Bonferroni-adjusted *p* values were calculated and reported. Statistical significance for the adjusted pairwise comparisons was set at *p* < 0.05. Effect sizes for the Kruskal–Wallis test were calculated using epsilon squared (ε^2^) and interpreted as negligible (<0.01), small (0.01–0.059), moderate (0.06–0.139), or large (≥0.14) [35]. Associations between balance outcomes and lower-limb symmetry indices or clinical variables were examined separately within each study group using Spearman’s rank correlation coefficient (ρ). To account for multiple testing across the 12 correlation coefficients reported in the study, *p* values were adjusted using the Benjamini–Hochberg false discovery rate (FDR) procedure.

To determine whether lower-limb symmetry indices explained additional variance in dynamic postural control after controlling for demographic characteristics and diagnostic group, hierarchical multiple linear regression analysis was performed using listwise deletion for missing data. Demographic variables (age, body mass index, and sex) were entered in the first block, diagnostic group (dummy-coded CAI and ACL groups, with healthy controls as the reference category) in the second block, and lower-limb symmetry indices (quadriceps LSI at 60°/s, hamstring LSI at 60°/s, dorsiflexor LSI at 60°/s, and plantarflexor LSI at 60°/s) in the third block. Sex was coded as 1 = male and 2 = female.

The regression model was specifically structured to examine the incremental contribution of LSI variables beyond demographic characteristics and diagnostic group; therefore, pain, physical activity, and quality-of-life measures were not included as additional covariates. The assumptions of linear regression were evaluated by examining linearity, normality and homoscedasticity of residuals, multicollinearity using variance inflation factor (VIF) and tolerance, independence of residuals using the Durbin–Watson statistic, and influential observations using Cook’s distance. A two-tailed *p* value of <0.05 was considered statistically significant; for the correlation analyses, statistical significance was determined using FDR-adjusted *p* values.

## 3. Results

### 3.1. Participant Characteristics

A total of 72 participants were included in the study, comprising 24 healthy controls, 24 individuals with CAI, and 24 individuals with ACL injury. There were no significant between-group differences in age or body mass index (both *p* > 0.05).

The control group consisted of 14 men (58.3%) and 10 women (41.7%), the CAI group of 12 men (50.0%) and 12 women (50.0%), and the ACL group of 15 men (62.5%) and 9 women (37.5%). All control participants were classified as healthy. All participants in both the CAI and ACL groups were classified as having chronic injuries (>6 months). Regarding surgical history, none of the healthy controls or participants in the CAI group had undergone surgery related to the lower extremities, whereas 12 participants (50.0%) in the ACL group had undergone ACL reconstruction.

### 3.2. Between-Group Comparisons

**Figure 1:**
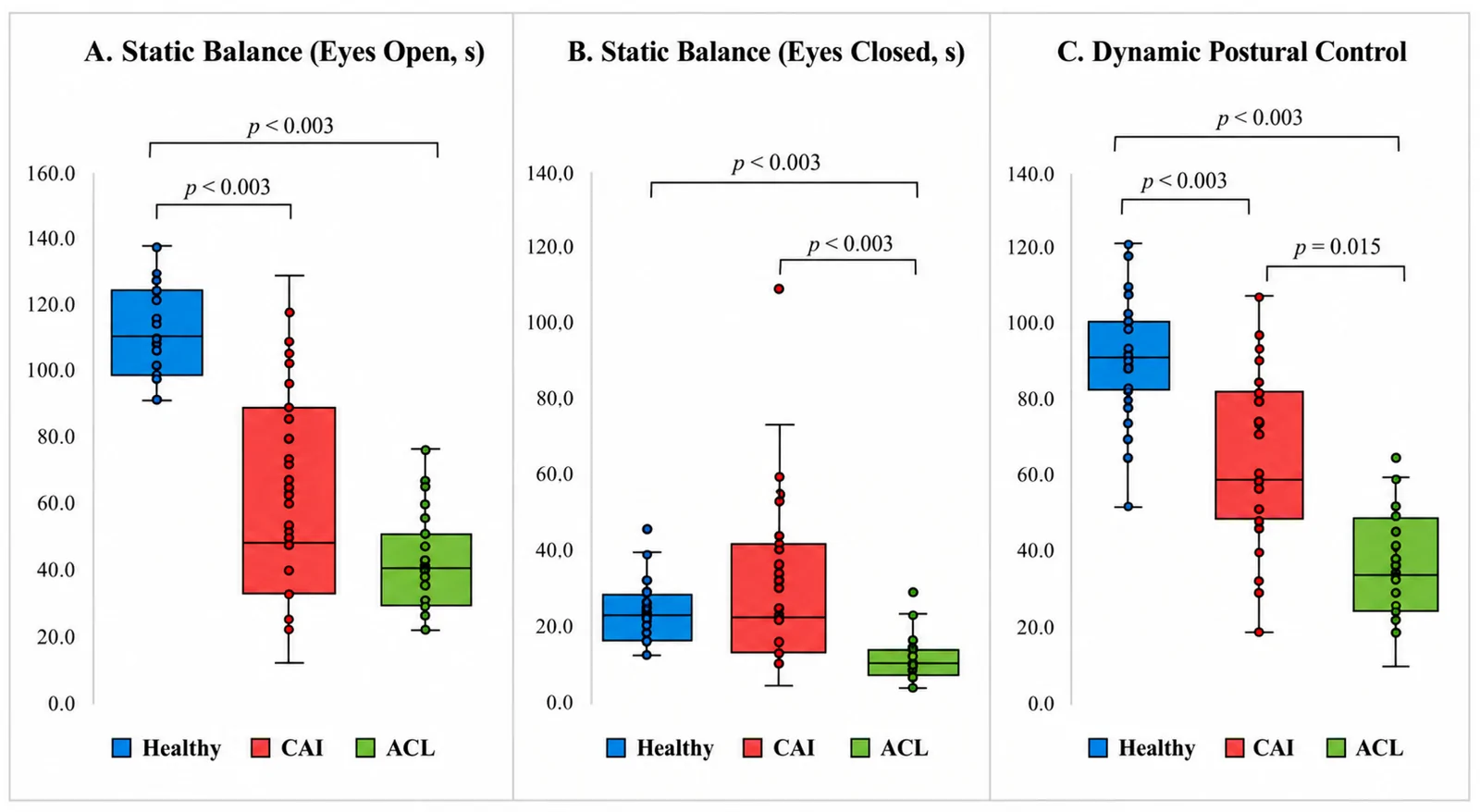
Comparison of static and dynamic postural control among healthy controls and individuals with chronic ankle instability (CAI) or anterior cruciate ligament (ACL) injury. Panel (**A**) shows static balance under eyes-open conditions, Panel (**B**) shows static balance under eyes-closed conditions, and Panel (**C**) shows dynamic postural control. Boxes represent the interquartile range (IQR), horizontal lines indicate the median, whiskers represent the minimum and maximum values, and circles represent individual participants. Higher static balance times under both eyes-open and eyes-closed conditions and higher dynamic postural-control scores indicate better performance. Only statistically significant pairwise comparisons are shown; displayed *p*-values are Bonferroni-adjusted.

Between-group comparisons and Bonferroni-adjusted pairwise comparisons are presented in Table 1. Significant overall group differences were observed for all balance outcomes (Figure 1), most limb symmetry indices, pain intensity, kinesiophobia, physical activity level, and patient-reported outcome measures (all *p* < 0.05), with effect sizes ranging from small to large. Pairwise comparisons showed higher eyes-open static balance in healthy controls than in both the CAI and ACL groups, with no significant difference between CAI and ACL. For eyes-closed static balance, the ACL group had lower values than both the healthy control and CAI groups, while the healthy control and CAI groups did not differ significantly. Dynamic postural control was higher in healthy controls than in both patient groups and higher in CAI than in ACL. The largest effect sizes were observed for SF-36 Physical Health Summary (ε^2^ = 0.644), dynamic postural control (ε^2^ = 0.591), static balance under eyes-open conditions (ε^2^ = 0.529), and pain intensity (ε^2^ = 0.508). In contrast, no significant overall group differences were found for age, body mass index, quadriceps LSI at 240°/s, or dorsiflexor LSI at 180°/s (*p* ≥ 0.05).

Limb-specific body-weight-normalized peak torque values are provided in Supplementary Table S1. Group effects were identified at 240°/s for quadriceps and at 180°/s for dorsiflexors and plantarflexors. Following Bonferroni adjustment, differences remained between controls and CAI for uninvolved-side dorsiflexors and both quadriceps sides; between controls and ACL for involved-side quadriceps; and between controls and CAI and between CAI and ACL for uninvolved-side plantarflexors.

### 3.3. Correlation Analyses

**Figure 2:**
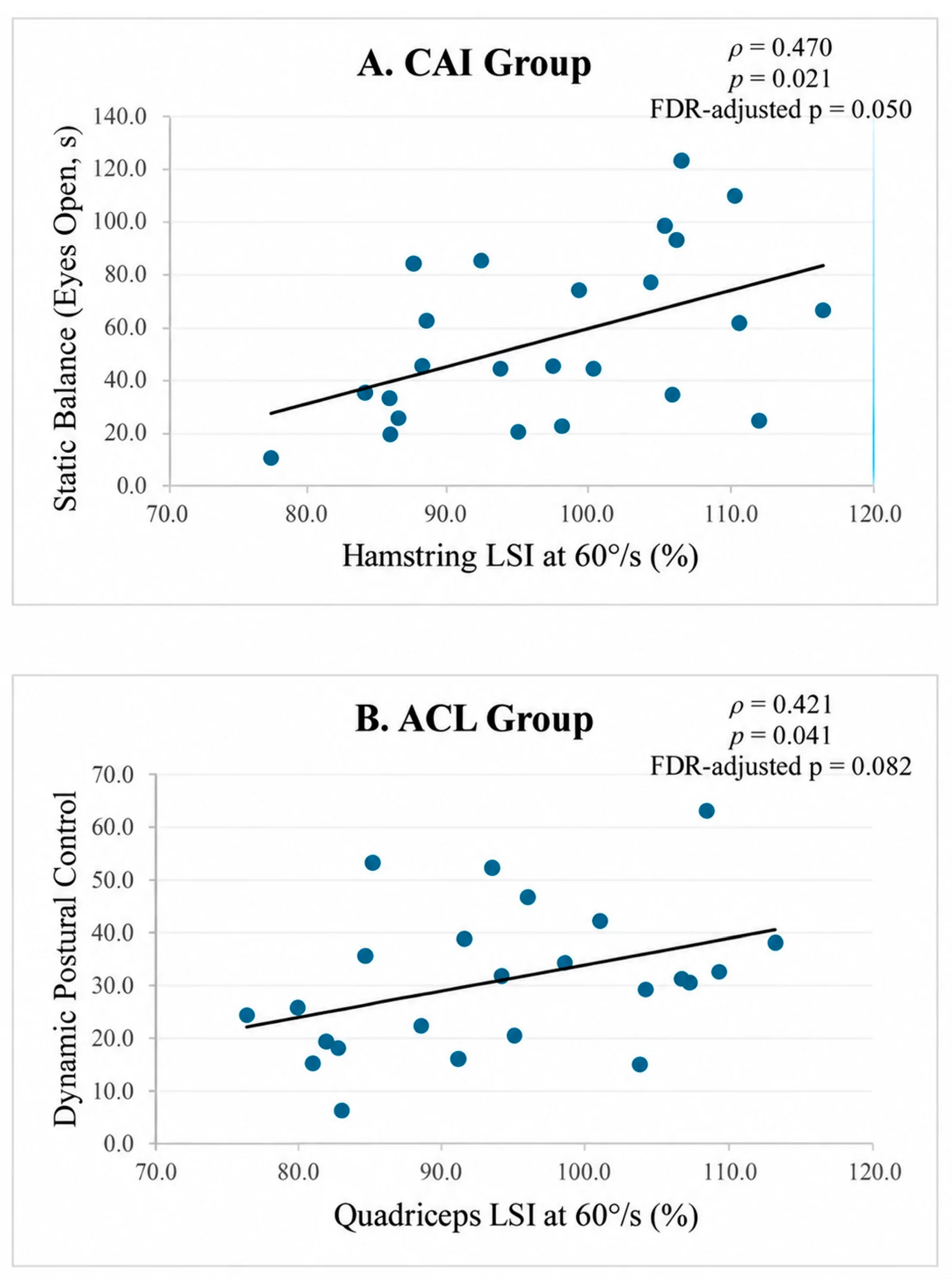
Scatter plots illustrate the associations observed in the unadjusted analyses between lower-limb muscle strength symmetry and postural control. (**A**) Association between hamstring limb symmetry index (LSI) at 60°/s and static balance under eyes-open conditions in individuals with chronic ankle instability (CAI). (**B**) Association between quadriceps limb symmetry index (LSI) at 60°/s and dynamic postural control in individuals with anterior cruciate ligament (ACL) injury. Associations were assessed using Spearman’s rank correlation. Neither association remained statistically significant after Benjamini–Hochberg false discovery rate correction. Solid fitted lines are shown for descriptive visualization only and do not represent the inferential analysis. Given the subgroup sample size (n = 24), these associations should be interpreted cautiously.

Spearman correlation analyses identified two nominal associations between lower-limb symmetry indices and balance outcomes in the unadjusted analyses (Figure 2). In the CAI group, hamstring LSI at 60°/s was moderately positively correlated with single-leg stance performance under eyes-open conditions (ρ = 0.470, unadjusted *p* = 0.021). In the ACL group, quadriceps LSI at 60°/s showed a moderate positive correlation with dynamic postural control (ρ = 0.421, unadjusted *p* = 0.041). However, neither association remained statistically significant after FDR correction (adjusted *p* = 0.0504 and 0.082, respectively). No other nominal associations between lower-limb symmetry indices and balance outcomes were observed in the healthy control group or either clinical group. Leave-one-out sensitivity analyses showed that the direction and magnitude of these two unadjusted associations remained reasonably stable, although statistical significance varied across some iterations, particularly in the ACL group.

Correlations between dynamic postural control and clinical variables are summarized in Table 2. After FDR correction, dynamic postural control remained negatively correlated with pain intensity in the CAI group (ρ = −0.652, adjusted *p* = 0.004). In the ACL group, dynamic postural control remained positively correlated with physical activity level (ρ = 0.583, adjusted *p* = 0.009), SF-36 physical health summary (ρ = 0.643, adjusted *p* = 0.004), and SF-36 mental health summary (ρ = 0.685, adjusted *p* = 0.003). No other clinical-variable correlations remained statistically significant after FDR correction.

### 3.4. Hierarchical Multiple Regression

Hierarchical multiple regression analysis was performed in the full sample (n = 72) to determine whether lower-limb symmetry indices explained additional variance in dynamic postural control after controlling for demographic characteristics and diagnostic group (Table 3, Panels A and B). As shown in Panel A, the first model, including age, body mass index, and sex, was not statistically significant (F(3, 68) = 2.208, R^2^ = 0.089, *p* = 0.095). The addition of diagnostic group in the second model significantly increased the explained variance in dynamic postural control (ΔR^2^ = 0.581, *p* < 0.001), accounting for the largest increase in explained variance across the hierarchical models; the overall model was statistically significant, F(5, 66) = 26.716, *p* < 0.001. In contrast, adding lower-limb symmetry indices in the final model did not significantly increase the explained variance (ΔR^2^ = 0.015, *p* = 0.583). The final model explained 68.4% of the variance in dynamic postural control (adjusted R^2^ = 0.638, F(9, 62) = 14.908, *p* < 0.001). 

As presented in Panel B, female sex was associated with a lower predicted dynamic postural-control score than male sex (B = −11.373, 95% CI −20.328 to −2.417, *p* = 0.014). Compared with healthy controls, both CAI (B = −30.879, 95% CI −43.300 to −18.459, *p* < 0.001) and ACL injury (B = −48.599, 95% CI −59.793 to −37.405, *p* < 0.001) were associated with lower predicted dynamic postural-control scores. None of the lower-limb symmetry indices explained additional variance after adjustment for demographic characteristics and diagnostic group. Regression assumptions were satisfied, with residual diagnostics indicating no substantial violations of normality, linearity, or homoscedasticity, and no evidence of multicollinearity (VIF = 1.05–2.46), influential observations (maximum Cook’s distance = 0.144), or autocorrelation of residuals (Durbin–Watson = 1.81). Pairwise correlations among the LSI variables included in the regression model were low (ρ = 0.066–0.179), further indicating no substantial intercorrelation among these predictors.

## 4. Discussion

The present study examined lower-limb muscle strength symmetry, postural control, and clinical characteristics in individuals with ACL injury, CAI, and healthy controls. The findings revealed distinct diagnosis-specific patterns in postural control and several strength-symmetry measures. Although selected LSI measures showed nominal associations with balance within the clinical groups, these associations did not remain significant after correction for multiple testing. Furthermore, lower-limb symmetry indices did not explain additional variance in dynamic postural control after demographic characteristics and diagnostic group were considered. These findings highlight the distinction between group-level differences in strength symmetry, within-group associations with balance, and the incremental contribution of LSI to postural control.

Postural-control impairments were observed in both clinical groups. Individuals with ACL injury and CAI demonstrated poorer eyes-open static balance and dynamic postural control than healthy controls, while dynamic postural control was additionally poorer in the ACL group than in the CAI group. This difference should be interpreted as a diagnosis-specific finding in the present sample rather than as evidence of universally greater impairment in ACL injury than in CAI, as factors such as activity level, surgical status, injury characteristics, and previous rehabilitation may have contributed to the observed pattern. Previous studies have similarly demonstrated impaired static and dynamic postural control in individuals with ACL injury and CAI [19,20]. More recent systematic evidence has also demonstrated deficits during static single-leg stance in individuals with CAI [36], while alterations in neuromuscular control during dynamic tasks have also been reported in this population [13]. These findings support the view that postural-control deficits in lower-extremity injuries may involve factors beyond muscle strength alone. Alterations in proprioceptive input, sensorimotor integration, neuromuscular activation, and joint-specific stabilization have been proposed as potential contributors [13,14], although these mechanisms were not directly assessed in the present study. Although ACL injury and CAI affect anatomically distinct joints, the postural-control deficits observed in both groups underscore the relevance of considering multiple sensorimotor components when interpreting balance performance.

Between-group differences in muscle strength symmetry showed diagnosis- and muscle-specific patterns. Compared with healthy controls, lower quadriceps LSI at 60°/s was observed in the ACL group, whereas plantarflexor LSI was particularly reduced in the CAI group. These patterns may be related to different consequences of the two conditions. Arthrogenic muscle inhibition and altered neural activation have been proposed as contributors to quadriceps dysfunction following ACL injury [14], whereas altered ankle sensorimotor function, proprioception, and neuromuscular control have been described in CAI [13]. Recent evidence has linked impaired ankle force sense with balance deficits in individuals with CAI [37]. This finding further supports the potential contribution of sensory function to postural control beyond maximal force-generating capacity. However, these mechanisms were not directly assessed in the present study and therefore cannot conclusively explain the observed LSI differences. In addition, the ACL group included both reconstructed and non-reconstructed participants, and all reconstructed participants received a hamstring tendon autograft. Graft-related donor-site effects may influence postoperative strength profiles and represent an additional source of variability when interpreting quadriceps and hamstring symmetry [38].

Despite these between-group differences in LSI, within-group associations between strength symmetry and postural control were not supported after correction for multiple testing. Nominal associations were observed between hamstring LSI and eyes-open static balance in the CAI group and between quadriceps LSI and dynamic postural control in the ACL group, but neither remained statistically significant after FDR correction. Similarly, although plantarflexor LSI was lower in the CAI group, it was not significantly associated with postural-control outcomes, illustrating that a between-group difference in strength symmetry does not necessarily translate into a within-group relationship with balance performance. Accordingly, the observed correlations should be regarded as exploratory rather than evidence of diagnosis-specific relationships between strength symmetry and postural control. Their clinical relevance also remains uncertain because clinically meaningful thresholds for these relationships were not evaluated. Recent intervention evidence indicates that rehabilitation incorporating neuromuscular and postural-control components can improve postural-control outcomes in ACL-reconstructed individuals [39]; however, the present cross-sectional findings cannot determine whether changes in quadriceps symmetry would produce corresponding changes in balance. Overall, the lack of consistent associations across muscle groups and balance conditions suggests that LSI derived from peak torque captures only one aspect of the factors potentially related to postural control.

The hierarchical regression analysis further clarified the contribution of strength symmetry to dynamic postural control. Demographic characteristics explained relatively little variance, whereas diagnostic group accounted for the largest increase in explained variance. Both CAI and ACL injury were associated with lower dynamic postural-control scores relative to healthy controls in the adjusted model, with the larger negative coefficient for the ACL group corresponding to the between-group pattern observed for dynamic postural control. The subsequent addition of quadriceps, hamstring, dorsiflexor, and plantarflexor LSI values did not significantly improve the model. Thus, the selected LSI measures did not provide additional explanatory value for dynamic postural control after demographic characteristics and diagnostic group were considered. This finding should not be interpreted as evidence that muscle strength is unimportant for postural control; rather, it indicates that peak-torque-based inter-limb symmetry did not explain additional variance in this sample. The associations of diagnostic group with dynamic postural control may encompass multiple injury-related characteristics not captured by LSI, including sensory, neuromuscular, structural, and functional factors, which were not comprehensively measured in the present study.

These findings also underscore important considerations when interpreting LSI. As a ratio-based measure, LSI is inherently dependent on the reference limb. A value approaching 100% indicates relative symmetry, whereas a value exceeding 100% indicates greater performance of the numerator limb; neither necessarily reflects normal absolute strength. Favorable symmetry may consequently coexist with bilateral strength deficits. After ACL reconstruction, reliance on the contralateral limb as a reference may therefore overestimate the extent of quadriceps restoration when compared with matched healthy controls [40]. Longitudinal changes in contralateral limb strength may also influence LSI over time [10]. To complement the symmetry indices, limb-specific body-weight-normalized peak torque values are provided in Supplementary Table S1. Nevertheless, the present LSI measures were derived specifically from isokinetic peak torque and therefore represent only one dimension of neuromuscular performance. Symmetry estimates may differ across assessment methods [41]. Furthermore, qualitative and quantitative characteristics of the isokinetic torque curve may provide information beyond peak torque alone [42]. In this context, Lemos et al. [43] investigated quadriceps force fluctuations during maximal isometric contraction in athletes with ACL injury and their association with lower-limb functional performance. Although their assessment approach differed from the isokinetic peak-torque measurements used in the present study, their findings further highlight the potential relevance of neuromuscular characteristics beyond maximal torque magnitude when evaluating lower-limb function. Other neuromuscular characteristics not assessed in the present study, including rate of force development, muscle activation, and force sense, may likewise provide information beyond peak-torque-based LSI and warrant further investigation in relation to postural control.

Clinical characteristics showed different relationships with dynamic postural control across the two injury groups. Between-group differences in TSK scores should be interpreted cautiously, as statistical significance does not necessarily indicate a clinically meaningful difference in kinesiophobia. Nevertheless, previous studies in individuals with chronic ankle instability have reported associations between kinesiophobia and altered postural control, including greater postural sway, greater visual reliance, and poorer dynamic balance performance [44,45]. In the CAI group, greater pain intensity was associated with poorer dynamic postural control, whereas physical activity and SF-36 scores were not significantly associated with balance performance. Previous evidence indicates that pain alleviation may improve balance control and lower-limb muscular coordination in individuals with CAI [46]. However, the present cross-sectional analysis cannot establish whether pain contributes to poorer postural control or whether greater functional impairment is accompanied by greater pain. These findings suggest that pain should be considered alongside mechanical and sensorimotor measures when characterizing functional status in CAI, without implying a causal relationship.

In the ACL group, dynamic postural control was positively associated with physical activity and both the physical and mental health scores of the SF-36, whereas no significant association with pain intensity was observed. These findings indicate that balance performance in this group covaried with broader aspects of activity and self-reported health rather than with pain alone. This pattern is consistent with previous evidence supporting the relevance of both physical and mental health dimensions when evaluating patient-reported health after ACL reconstruction [47]. However, the direction of these relationships cannot be determined from the present cross-sectional data. Greater activity and better perceived health may accompany better postural control, but the temporal direction of these associations remains unknown. Interpretation should also account for the clinical heterogeneity of the ACL group, particularly with respect to reconstruction status and the unavailable exact time since reconstruction. The observed associations should therefore be interpreted as characteristics of this sample rather than as general relationships applicable to all individuals with ACL injury.

Collectively, the findings support a multidimensional interpretation of postural control following lower-extremity injury. Group differences in strength symmetry, balance performance, and patient-related characteristics did not show a uniform pattern across ACL injury and CAI, suggesting that these measures provide complementary rather than interchangeable information. From a clinical assessment perspective, favorable inter-limb symmetry should therefore not be assumed to indicate normalized postural control. Our findings support considering limb-specific strength alongside symmetry indices, static and dynamic balance performance, functional measures, and relevant patient-reported outcomes when characterizing functional status. These diagnosis-specific patterns may help inform comprehensive assessment but should not be interpreted as evidence for particular treatment strategies, because no rehabilitation intervention was evaluated.

### Strengths and Limitations

Nevertheless, several limitations should be considered. First, the cross-sectional design precludes conclusions regarding causal or temporal relationships between lower-limb muscle strength symmetry and postural control. The study was also registered after participant enrollment had commenced. Second, the relatively modest sample size, single-center recruitment, and inclusion of only young adults may limit generalizability to older adults, elite athletes, individuals with acute injuries, or patients at different stages of rehabilitation. At the achieved sample size (n = 72), the study was sensitive to detect between-group effects of approximately f = 0.374 (partial η^2^ ≈ 0.123) at 80% power and α = 0.05. Thus, non-significant comparisons should not be interpreted as evidence of no between-group difference, particularly for effects below this threshold. For the hierarchical regression, sensitivity analysis indicated that the incremental LSI block had 80% power to detect an effect size of f^2^ ≥ 0.179 at α = 0.05. The observed effect was smaller (ΔR^2^ = 0.015; f^2^ ≈ 0.047), and a smaller contribution of LSI therefore cannot be excluded. The number of predictors relative to the sample size also increased the risk of model overfitting, requiring confirmation in larger independent samples. The model was designed specifically to examine the incremental contribution of LSI beyond demographic characteristics and diagnostic group and should not be interpreted as identifying all factors associated with postural control. Clinical factors such as pain and physical activity may also contribute to, or potentially mediate, the association between diagnostic status and postural control; however, mediation was not examined.

Third, previous rehabilitation was not standardized across participants. Detailed information on its timing, duration, frequency, and content was not recorded, preventing evaluation of its potential influence on the outcomes. Time since diagnosis was recorded categorically within the predefined 6–18-month eligibility interval rather than as an exact continuous value, precluding calculation of mean or median duration. The ACL group included equal numbers of reconstructed and non-reconstructed participants (n = 12 each); however, the small subgroup sizes and unavailable information on the exact time since reconstruction precluded a meaningful subgroup sensitivity analysis. This heterogeneity should be considered when interpreting the findings. Patients and healthy controls were also recruited from different sources, potentially introducing selection-related differences between groups.

Fourth, LSI is a ratio-based measure that may mask bilateral strength deficits and should therefore be interpreted alongside limb-specific strength values. Although limb-specific body-weight-normalized peak torque values are provided in Supplementary Table S1, lower-limb strength symmetry was quantified using peak torque at 60°/s, which may not fully capture muscle performance across different contraction velocities or during functional and sport-specific activities. Fixed testing orders were used during both static balance and isokinetic assessments. The uninvolved limb was assessed before the involved limb in the clinical groups, whereas the dominant limb was assessed before the non-dominant limb in healthy controls. In static balance testing, the eyes-open condition also preceded the eyes-closed condition. These fixed sequences may have introduced order, learning, or fatigue effects, although standardized rest intervals were provided to minimize fatigue. Direct measures of proprioception, joint laxity, muscle activation, and sensory integration were not included, limiting assessment of their potential contributions to postural control. Assessor blinding was not ensured, which may have introduced measurement bias. Finally, the subgroup correlation analyses (n = 24 per group) had limited sensitivity to detect small-to-moderate associations, with a minimum detectable correlation of approximately |ρ| = 0.55 at 80% power and α = 0.05. Although FDR correction was applied for multiple testing, these analyses remain exploratory and should be interpreted cautiously.

## 5. Conclusions

Lower-limb muscle strength symmetry did not explain additional variance in dynamic postural control after adjustment for demographic characteristics and diagnostic group. These findings support a multidimensional approach to characterizing postural control in individuals with lower-extremity injuries. Lower-limb muscle strength symmetry should therefore be considered alongside objective postural control measures and patient-reported outcomes rather than in isolation. Future longitudinal studies are warranted to determine whether changes in lower-limb muscle strength symmetry are associated with changes in postural control across different lower-extremity injury populations.

## Figures and Tables

**Table 1:** Participant characteristics and between-group comparisons.

Variables	Control (n = 24) Median [IQR]	CAI (n = 24) Median [IQR]	ACL (n = 24) Median [IQR]	H	*p*	ε^2^	Post Hoc Comparisons (Bonferroni-Adjusted *p*)
Age (years)	28.50 [24.50–33.75]	30.00 [25.00–37.75]	30.00 [24.25–32.75]	1.035	0.596	0.000	**-**
BMI (kg/m^2^)	24.75 [22.85–26.75]	24.50 [20.85–26.60]	23.00 [22.00–24.00]	4.396	0.111	0.035	**-**
**Balance Outcomes**							
Static Balance EO (s)	106.50 [90.50–116.00]	45.00 [27.00–82.25]	40.00 [26.50–50.00]	38.514	<0.001 *	0.529	Control vs. CAI: **<0.003 ^§^** Control vs. ACL: **<0.003 ^§^** CAI vs. ACL: 0.810
Static Balance EC (s)	21.50 [16.25–25.00]	20.50 [12.75–40.50]	9.30 [8.00–12.00]	29.594	<0.001 *	0.400	Control vs. CAI: 1.000 Control vs. ACL: **<0.003 ^§^** CAI vs. ACL: **<0.003 ^§^**
Dynamic Postural Control	84.34 [69.51–90.41]	52.25 [25.77–64.23]	30.68 [19.43–38.53]	42.808	<0.001 *	0.591	Control vs. CAI: **<0.003 ^§^** Control vs. ACL: **<0.003 ^§^** CAI vs. ACL: **0.015 ^§^**
**Inter-Limb Symmetry Indices (%)**							
Quadriceps LSI (60°/s)	103.37 [100.92–106.05]	97.51 [86.81–108.01]	93.84 [83.41–104.11]	8.078	0.018 *	0.088	Control vs. CAI: 0.381 Control vs. ACL: **0.009 ^§^** CAI vs. ACL: 0.909
Quadriceps LSI (240°/s)	101.94 [98.26–106.50]	97.83 [90.26–104.60]	95.77 [90.81–101.12]	4.787	0.091	0.040	–
Hamstring LSI (60°/s)	106.22 [99.48–111.50]	97.46 [87.33–105.81]	91.52 [88.64–96.47]	14.616	0.001 *	0.183	Control vs. CAI: **0.021 ^§^** Control vs. ACL: **<0.003 ^§^** CAI vs. ACL: 0.708
Hamstring LSI (240°/s)	105.61 [101.35–123.04]	98.82 [88.47–109.87]	89.44 [87.41–97.10]	19.505	<0.001 *	0.254	Control vs. CAI: **0.009 ^§^** Control vs. ACL: **<0.003 ^§^** CAI vs. ACL: 0.243
Dorsiflexor LSI (60°/s)	105.88 [91.75–118.41]	92.45 [78.84–105.17]	89.64 [81.82–107.57]	8.118	0.017 *	0.089	Control vs. CAI: **0.039 ^§^** Control vs. ACL: **0.048 ^§^** CAI vs. ACL: 1.000
Dorsiflexor LSI (180°/s)	105.83 [90.00–111.32]	89.88 [85.92–95.87]	104.27 [91.25–111.00]	6.009	0.050	0.058	-
Plantarflexor LSI (60°/s)	103.65 [100.42–107.14]	90.00 [81.25–94.05]	96.15 [91.67–102.12]	27.687	<0.001 *	0.372	Control vs. CAI: **<0.003 ^§^** Control vs. ACL: **0.030 ^§^** CAI vs. ACL: **<0.003 ^§^**
Plantarflexor LSI (180°/s)	104.51 [83.82–109.67]	92.77 [82.20–97.16]	104.45 [94.50–110.93]	12.024	0.002 *	0.145	Control vs. CAI: **0.039 ^§^** Control vs. ACL: 1.000 CAI vs. ACL: **0.003 ^§^**
**Clinical Outcomes**							
Pain Intensity (cm)	0.00 [0.00–0.00]	2.00 [0.00–3.75]	3.00 [2.00–3.00]	37.034	<0.001 *	0.508	Control vs. CAI: **<0.003 ^§^** Control vs. ACL: **<0.003 ^§^** CAI vs. ACL: 0.522
Kinesiophobia	40.00 [37.00–44.00]	43.00 [40.00–48.50]	46.00 [43.00–49.00]	16.898	<0.001 *	0.216	Control vs. CAI: 0.081 Control vs. ACL: **<0.003 ^§^** CAI vs. ACL: 0.153
Physical Activity Level (MET-min/week)	1315.50 [616.50–3113.33]	787.00 [676.00–979.00]	2779.00 [2323.50–3709.50]	29.043	<0.001 *	0.392	Control vs. CAI: 0.213 Control vs. ACL: **0.015 ^§^** CAI vs. ACL: **<0.003 ^§^**
SF-36 Physical Health Summary	83.13 [67.50–90.00]	64.69 [59.53–70.00]	47.13 [34.06–55.50]	46.439	<0.001 *	0.644	Control vs. CAI: **<0.003 ^§^** Control vs. ACL: **<0.003 ^§^** CAI vs. ACL: **<0.003 ^§^**
SF-36 Mental Health Summary	83.19 [61.28–86.31]	65.63 [57.31–73.56]	53.75 [42.63–60.94]	26.767	<0.001 *	0.359	Control vs. CAI: **0.012 ^§^** Control vs. ACL: **<0.003 ^§^** CAI vs. ACL: **<0.003 ^§^**

CAI: chronic ankle instability; ACL: anterior cruciate ligament injury; LSI: limb symmetry index; EO: eyes open; EC: eyes closed; SF-36: Short Form-36 Health Survey. Effect size (ε^2^) interpretation: <0.01, negligible; 0.01–0.059, small; 0.06–0.139, moderate; ≥0.14, large; * *p* < 0.05 for the Kruskal–Wallis test; ^§^: statistically significant adjusted *p* < 0.05.

**Table 2:** Spearman correlations of dynamic postural control with clinical characteristics in the patient groups.

Variable	CAI (n = 24)				ACL (n = 24)			
	ρ	95% CI	Raw *p*	FDR *p*	ρ	95% CI	Raw *p*	FDR *p*
Pain Intensity (cm)	**−0.652**	−0.869 to −0.297	0.001 *	0.004 *	−0.015	−0.382 to 0.344	0.943	0.943
Kinesiophobia	−0.258	−0.640 to 0.177	0.223	0.297	−0.362	−0.716 to 0.093	0.082	0.141
Physical Activity Level (MET-min/week)	0.084	−0.366 to 0.496	0.695	0.758	**0.583**	0.228 to 0.838	0.003 *	0.009 *
SF-36 Physical Health Summary	0.117	−0.299 to 0.496	0.585	0.702	**0.643**	0.307 to 0.872	0.001 *	0.004 *
SF-36 Mental Health Summary	0.299	−0.147 to 0.664	0.156	0.234	**0.685**	0.406 to 0.847	<0.001 *	0.003 *

CAI: chronic ankle instability; ACL: anterior cruciate ligament injury; SF-36: Short Form-36 Health Survey. Values are Spearman’s ρ with BCa 95% confidence intervals based on 5000 bootstrap samples. Raw and Benjamini–Hochberg false discovery rate (FDR)-adjusted *p* values are reported. * FDR-adjusted *p* < 0.05.

**Table 3:** Hierarchical multiple regression predicting dynamic postural control.

**Panel A. Model Summary**								
**Model**	**Predictors Entered**	**R^2^**		**Adjusted R^2^**	**ΔR^2^**	**ΔF**	***p* for ΔF**	**Overall F (df), *p***
**1**	Age, BMI, Sex	0.089		0.049	0.089	2.208	0.095	F (3, 68) = 2.208, *p* = 0.095
**2**	+ Diagnostic group (CAI, ACL)	0.669		0.644	0.581	57.932	<0.001 *	F (5, 66) = 26.716, *p* < 0.001 *
**3**	+ Lower-limb symmetry indices	0.684		0.638	0.015	0.718	0.583	F (9, 62) = 14.908, *p* < 0.001 *
**Panel B. Final Regression Coefficients (Model 3)**								
**Predictor**		**B**	**SE**	**β**	**95% CI**		***p***	**VIF**
Age (years)		−0.144	0.328	−0.032	−0.800 to 0.512		0.662	1.053
BMI (kg/m^2^)		0.038	0.723	0.004	−1.406 to 1.483		0.958	1.390
Sex		−11.373	4.480	−0.215	−20.328 to −2.417		0.014 *	1.409
CAI		−30.879	6.214	−0.556	−43.300 to −18.459		<0.001 *	2.456
ACL		−48.599	5.600	−0.875	−59.793 to −37.405		<0.001 *	1.995
Quadriceps LSI (%) (60°/s)		0.062	0.172	0.027	−0.282 to 0.406		0.721	1.137
Hamstring LSI (%) (60°/s)		0.209	0.173	0.096	−0.136 to 0.554		0.231	1.242
Dorsiflexor LSI (%) (60°/s)		−0.101	0.092	−0.085	−0.284 to 0.082		0.274	1.177
Plantarflexor LSI (%) (60°/s)		0.001	0.211	0.000	−0.422 to 0.424		0.996	1.600

BMI: body mass index; CAI: chronic ankle instability; ACL: anterior cruciate ligament injury; LSI: limb symmetry index; CI: confidence interval; VIF: variance inflation factor. N = 72. Sex was coded as 1 = male and 2 = female. Diagnostic group was dummy-coded with healthy controls as the reference category (CAI: 0 = no, 1 = yes; ACL: 0 = no, 1 = yes). * *p* < 0.05.

## Data Availability

The data that support the findings of this study are available from the corresponding author, upon reasonable request.

## Supplementary Materials

The following supporting information can be downloaded at: https://www.mdpi.com/article/10.3390/jcm15207979/s1, Table S1: Limb-specific body-weight-normalized peak torque values across isokinetic testing velocities.
