# Supplementary material for: Lower-Limb Strength Symmetry, Postural Control, and Clinical Characteristics in Individuals with ACL Injury and Chronic Ankle Instability
Source: J Clin Med. 2026 Oct 15;15(20):7979. doi: 10.3390/jcm15207979 (PMC13706059; doi:10.3390/jcm15207979)
Supplement: Supplementary file 1 [file jcm-15-07979-s001.zip › jcm-4521768-supplementary.pdf]

**Supplementary Table S1. Limb-specific body-weight-normalized peak torque values across isokinetic testing velocities**

| Muscle group | Angular velocity | Healthy controls (n=24) Mean ± SD | | CAI (n=24) Mean ± SD | | ACL injury (n=24) Mean ± SD | | $p^1$ | $p^2$ |
|---|---|---|---|---|---|---|---|---|---|
| | | Dominant | Non-dominant | Involved | Uninvolved | Involved | Uninvolved | | |
| Quadriceps | 60°/s | 129.25 ± 30.70 | 124.79 ± 28.10 | 124.83 ± 23.03 | 129.42 ± 29.92 | 137.63 ± 31.73 | 148.88 ± 41.57 | 0.506 | 0.099 |
| | 240°/s | 90.04 ± 9.72 | 90.17 ± 13.17 | 76.25 ± 14.85 | 78.88 ± 16.07 | 77.42 ± 13.27 | 81.04 ± 14.36 | **0.001*** | **0.017*** |
| Hamstrings | 60°/s | 102.17 ± 16.38 | 97.25 ± 14.73 | 98.46 ± 16.24 | 102.04 ± 17.58 | 101.79 ± 25.72 | 110.08 ± 29.64 | 0.610 | 0.477 |
| | 240°/s | 72.75 ± 12.50 | 66.71 ± 14.25 | 68.83 ± 12.70 | 70.63 ± 14.01 | 67.63 ± 8.49 | 74.17 ± 11.82 | 0.323 | 0.167 |
| Dorsiflexors | 60°/s | 27.29 ± 11.50 | 25.75 ± 11.00 | 28.96 ± 12.94 | 32.04 ± 14.85 | 20.96 ± 4.19 | 23.58 ± 5.61 | 0.099 | 0.153 |
| | 180°/s | 17.88 ± 7.99 | 17.63 ± 8.41 | 23.63 ± 12.29 | 25.38 ± 12.76 | 18.30 ± 3.94 | 18.42 ± 5.23 | 0.108 | **0.018*** |
| Plantarflexors | 60°/s | 40.88 ± 20.04 | 39.33 ± 18.98 | 37.63 ± 13.80 | 42.25 ± 13.26 | 43.29 ± 7.39 | 43.92 ± 8.27 | 0.214 | 0.848 |
| | 180°/s | 30.04 ± 13.16 | 30.46 ± 12.37 | 35.29 ± 12.71 | 39.54 ± 11.47 | 31.57 ± 3.63 | 31.71 ± 10.11 | **0.045*** | **0.002*** |

**Note:** Values are presented as mean ± SD. Peak torque was normalized to body weight and expressed as Peak Torque / Body Weight (%). $p^1$ represents the Kruskal–Wallis comparison of the dominant limb in healthy controls with the involved limb in the CAI and ACL groups; $p^2$ represents the comparison of the non-dominant limb in healthy controls with the uninvolved limb in the CAI and ACL groups. $p<0.05$. Significant overall Kruskal–Wallis tests were followed by pairwise Mann–Whitney U tests with Bonferroni adjustment. Bonferroni-adjusted pairwise comparisons were significant for dorsiflexor strength at 180°/s on the uninvolved/non-dominant side (Control–CAI, p=.024), plantarflexor strength at 180°/s on the uninvolved/non-dominant side (Control–CAI, p=.006; CAI–ACL, p=.009), and quadriceps strength at 240°/s on the involved/dominant side (Control–CAI, p=.003; Control–ACL, p=.006) and uninvolved/non-dominant side (Control–CAI, p=.021). No pairwise comparison remained significant for plantarflexor strength at 180°/s on the involved/dominant side after Bonferroni adjustment. CAI, chronic ankle instability; ACL, anterior cruciate ligament; SD, standard deviation.
